# Telemedicine and eHealth Literacy in the Era of COVID-19: A Cross-Sectional Study in a Peripheral Clinic in Israel

**DOI:** 10.3390/ijerph18189556

**Published:** 2021-09-10

**Authors:** Keren Dopelt, Nofar Avni, Yana Haimov-Sadikov, Iris Golan, Nadav Davidovitch

**Affiliations:** 1 Department of Public Health, Ashkelon Academic College, 12 Ben Tzvi St., Ashkelon 78211, Israel; avninofar@edu.aac.ac.il (N.A.); yanah@edu.aac.ac.il (Y.H.-S.); iriska@edu.aac.ac.il (I.G.); 2 Department of Health Policy and Management, School of Public Health, Faculty of Health Sciences, Ben-Gurion University of the Negev, Beer Sheva 84105, Israel; nadavd@bgu.ac.il

**Keywords:** telemedicine, eHealth literacy, internet, access, periphery, COVID-19

## Abstract

The COVID-19 pandemic mandating isolation, quarantine, and social distancing has accelerated and expanded the use of telemedicine. This study examines the extent of the use of telemedicine and the relationship between eHealth literacy and satisfaction with using telemedicine during the pandemic. A total of 156 participants from a clinic in a peripheral community in southern Israel completed an online questionnaire. We found that 85% knew how to use the internet for health information, but only one third felt safe using it to make health decisions. Furthermore, 93% used the internet for technical needs, such as renewing prescriptions or making a doctor’s appointment. Even lower use for telemedicine was found (38%) for consultation or treatment sessions. A positive association was found between eHealth literacy and satisfaction variables with using telemedicine (r_p_ = 0.39, *p* < 0.001). Although respondents understood the benefits of telemedicine, they were not satisfied nor interested in online sessions after the epidemic’s end, preferring a meeting involving personal interaction. Young people and academics benefit more from telemedicine, thereby creating usage gaps and potentially increasing existing inequality. We recommend developing intervention programs, especially among vulnerable populations, to strengthen eHealth literacy and remove barriers causing skepticism about the use of telemedicine during and after the pandemic.

## 1. Introduction

The COVID-19 pandemic has accelerated and expanded the use of telemedicine throughout the world. The need for and advantages of telemedicine is most evident during periods when people are required to remain in isolation or lockdowns, or maintain social distancing to reduce infection. Essential health services must continue to be provided within communities even while the health system is preoccupied with contending with the virus in order avoid causing harm to the general public health or contribute to any deterioration in patients’ medical conditions. Telemedicine makes it possible to guarantee continued medical treatment while still reducing the transmission of the virus [1]. Scientific societies around the world recommended the use of tele-health even before COVID-19, adjusted for patients, their families, healthcare professionals, and organizations.

Studies have shown that an online session with a physician may be effective even in relatively complicated medical cases. Thus, for example, a study conducted several years ago found a number of advantages to treatment of chronic congestive heart failure by telemedicine, including reduced hospitalization time and fewer cases of mortality [2]. This involved a saving of both time and costs for the patients [3], and access for the elderly, people living in geographically remote areas, and those with disabilities [4], as well as the prevention of contracting infectious diseases for both patients and staff [5]. The disadvantages of using telemedicine relate to medical confidentiality and concern over hacking information systems, the inability to provide lifesaving care or to conduct certain tests immediately, and the inability to undergo a physical checkup, as well as obstacles related to clinician–patient relations [6]. The use of telemedicine depends, to a large extent, on the level of eHealth literacy of the specific individual [7].

The current study seeks to shed light on the relationship between age, eHealth literacy, and satisfaction with telemedicine in the context of its use in geographically outlying areas during the COVID-19 pandemic. For this purpose, 156 participants from a clinic in a peripheral community in southern Israel completed an online questionnaire measuring healthcare related actions conducted over the internet, eHealth literacy, and satisfaction with telemedicine.

The study will contribute to the understanding of the relationships between age, eHealth literacy, and satisfaction with telemedicine, thereby pointing to possible difficulties in using telemedicine in different populations when eHealth literacy and satisfaction are low. In practical terms, if the research hypotheses are confirmed, we can recommend an intervention program to policymakers that will increase the level of eHealth literacy among a variety of population groups in Israel, in order to give them more confidence in using telemedicine.

In the next part of the paper, we will explain the definition of eHealth literacy, and we will present studies linking between the research variables. Then, we will detail the research method and results. Finally, we will discuss the results and present conclusions and recommendations.

## 2. Literature Review

### eHealth Literacy

Electronic health (or eHealth) literacy is defined as the ability to search, locate, and understand health information from electronic sources, to evaluate the quality of the information, and to apply it in order to address a given health problem or to improve a health condition [8]. People with a good level of eHealth literacy receive more healthcare information, enjoy more varied sources of information, are better equipped to evaluate the quality of the information, and report a much greater volume of usage results [9]. eHealth literacy poses a challenge to various population groups, such as immigrants, ethnic minorities, the elderly, low-income individuals, and those living in areas without home broadband access [10]. Dobrusin et al. [11] found that, during the COVID-19 pandemic, the use of telemedicine increased dramatically, and younger patients tended to be more satisfied with virtual treatment sessions than older patients. In other words, it is precisely those people who are in greater need of healthcare services who found it more difficult to use telemedicine; consequently, they expressed a lower degree of satisfaction. People with limited eHealth literacy are likely to have less access to reliable materials based on internet healthcare education [12,13]. Indeed, they are at a higher risk of negative health results compared with patients with a higher degree of eHealth literacy [14]. Low eHealth literacy is related to a lifestyle that is not conducive to improving health, difficulty in navigating the maze of the health system, and under-use of healthcare services [15,16], as well as a low response to treatment using medication, hospitalization, and a greater risk of mortality [17,18]. Studies have shown that people with a high degree of eHealth literacy who suffer from chronic diseases, such as diabetes and hypertension, tend to monitor and manage their diseases more competently, are more satisfied with the telemedicine services, and respond faster to changes that might adversely affect their situation, thereby improving their health [19,20,21].

The COVID-19 pandemic accelerated a number of new processes, including the development of telemedicine. Clinician–patient meetings via the internet were an inevitable consequence of the need for social distancing. Generally, people without access to the internet or lacking the requisite technological skills are likely to encounter difficulty in obtaining medical services [22]. The current study seeks to shed light on the relationship between age, eHealth literacy, and satisfaction with telemedicine in the context of its use in geographically outlying areas during the COVID-19 pandemic. We hypothesized that negative correlations would be found between age and eHealth literacy, and between age and satisfaction with telemedicine. In addition, we hypothesized that a positive correlation would be found between eHealth literacy and satisfaction with telemedicine.

## 3. Materials and Methods

A cross-sectional study was conducted using an online questionnaire, which enables ease of data gathering in the era of COVID-19.

### 3.1. Participants and Process

In the current study, 156 people who belong to a primary medicine clinic in the southern peripheral area of Israel participated, all of whom were above the age of 18. The survey was conducted at the request of the clinic manager who wished to ensure that the patients had succeeded in attaining sufficient eHealth literacy and were able to use the telemedicine services during the pandemic. The clinic has 412 members over the age of 18. Approval for the study was received from the Ashkelon Academic College Ethics Committee (Approval No. #28-2020). The questionnaires were programmed using the Qualtrics survey software platform, London, UK. The link to the survey was sent via text message from the clinic on 17 December 2020, with a reminder sent a week later. On 31 December 2020, the survey was closed on the software platform. According to the software data, the average response time to complete the questionnaire was four minutes. There were 187 entries into the survey, and 156 participants completed the questionnaire (83% of the total number of entries into the survey, 38% of the total number of adult patients at the clinic).

### 3.2. The Study Tools

An online, closed-ended, anonymous, self-administered questionnaire (Appendix A) was used in the study. The questionnaires were translated from English by the authors. Initially, the questionnaire was translated from English into Hebrew, then back-translated from Hebrew into English, and finally back into Hebrew, and the different translation versions were then compared to ensure the reliability of the translation. In the second stage, a pilot test was conducted among ten people to ensure that the questions were readily comprehensible; the questionnaire was amended in accordance with the subsequent comments. Finally, the questionnaire was approved for content validity by three telemedicine and public health experts. In their report, the experts wrote that the questions were related to the subject matter of the variables and were appropriate for Israeli culture, and they proposed adding two questions related to the COVID-19 pandemic, as detailed in category number 3 below. The questionnaire contained 34 questions according to the following categories:

1. Demographic data—gender, date of birth, level of education, country of birth, year of aliyah, and having health-related apps installed on their mobile phones.
2. Healthcare-related actions conducted over the internet during the last six months, involving eight kinds of actions. This includes checking test results, scheduling a doctor’s appointment or an online treatment session, and attending medical forums for consultation. The participants were required to provide yes/no answers for each action.
3. eHealth literacy—seven questions, the first five of which were taken from Norman & Skinner [23], and the last two dealt with the COVID-19 pandemic, were added by the authors on the recommendation of the experts. The examinees were asked to write down to what extent they agreed with each statement in the questionnaire on a scale ranging from 1 (not at all) to 5 (to a very large extent). For example, regarding how to use the internet to respond to health-related questions, how to find useful information about healthcare and the COVID-19 pandemic on the internet: consume more healthcare-related information on the internet since the outbreak of the COVID-19 pandemic, and how to look for information on the internet about the COVID-19 vaccine. The variable was built by calculating the average for each examinee. The average ranged between 1–5, with a higher score indicative of a higher level of eHealth literacy. Internal reliability was α = 0.80.
4. Satisfaction with telemedicine—ten questions taken from Parmanto, Lewis, Graham & Bertolet [24]. The examinees were asked to write down to what extent they agreed with each statement in the questionnaire on a scale ranging from 1 (not at all) to 5 (to a very large extent), and an option was also given to write down 6 (irrelevant). For example, “I am satisfied with my experience of a treatment session with medical staff via the internet”, and “I will continue to use telemedicine services for treatment sessions even after the pandemic.” The variable was built by calculating the average for each examinee, excluding the option of “irrelevant.” Reversing scales was carried out for the question: “I tried to enter an online treatment session but without success.” The average ranged between 1–5, whereby a higher score was indicative of a greater use of telemedicine. Internal reliability was α = 0.89.

### 3.3. Data Processing

The data were processed with SPSS V. 25 software. The distribution of responses to questions was analyzed after combining categories as follows: answers 1 and 2 were combined into the category ‘small extent,’ answer 3 remained ‘moderate extent’ and answers 4 and 5 were combined into the category ‘large extent’. Preliminary analysis indicated a normal distribution of variables, so the correlations between the variables were examined using the Pearson correlation coefficient, and gender differences were examined using *t*-tests for independent samples. The age variable was not normally distributed, thefore we used the non-parametric Spearman correlation. Finally, a (multiple) linear regression model was constructed to examine those variables that best forecast satisfaction with telemedicine.

## 4. Results

### 4.1. Description of the Sample Characteristics

One hundred and fifty-six participants completed the survey. The age range was between 19–76, and the average age was 34 (SD = 16.48). The time elapsed since immigrating to Israel for those participants not born in Israel (*n* = 31, 20%) ranged between 10–61, with the average time being 32 (SD = 10.54). See sample characteristics in Table 1.

### 4.2. eHealth Literacy

It was found that 85% of the respondents had significant knowledge about how to use the internet to reply to health-related questions. Seventy-eight percent had significant knowledge about where to find useful healthcare information on the internet. Two thirds knew how to use the healthcare information they find on the internet. However, only 60% of the respondents had significant knowledge about how to evaluate the healthcare information they find on the internet, and a mere 36% felt confident about using the information on the internet to make health-related decisions. Forty-one percent now consume significantly more healthcare information on the internet since the outbreak of the COVID-19 pandemic, and one third reported that they searched for material on the internet on the COVID-19 vaccine to a large extent. In order to construct the eHealth literacy variable, the average of all the answers of each participant was calculated. The overall average was 3.58 (SD = 0.72).

### 4.3. Satisfaction with Telemedicine

Generally, 59% were satisfied to a large extent with the telemedicine services and 23% to a moderate extent. A further finding was that 81% believed that telemedicine saves significant time and waiting in line, 80% thought that telemedicine constitutes an excellent solution for the COVID-19 era, 70% felt that the apps were easy to use, 68% reported that telemedicine improved their access to the healthcare services, while 61% believed that telemedicine is appropriate for their healthcare needs. Close to one half (45%) assumed that they would continue to use telemedicine services for their treatment sessions even after the pandemic. About one third of the overall sample (36%, representing 53% of all the respondents who attended a treatment session on the internet) were satisfied with their experiences with a treatment session on the internet with healthcare staff. However, only one quarter (representing 38% of all the respondents who attended a treatment session on the internet) thought that the visits held online were identical in quality to in-person visits.

### 4.4. The Relationships between the Variables

The relationships were examined using the Pearson or Spearman correlation coefficient. No correlation was found between age and eHealth literacy (*p* = 0.67). However, a negative correlation of moderate significance was found between age and satisfaction with telemedicine (r_s_ = −0.19, *p* < 0.05). In addition, a positive correlation of high significance was found between eHealth literacy and satisfaction with telemedicine (r_p_ = 0.39, *p* < 0.001) Namely, the more an examinee was eHealth literate, the higher his/her satisfaction with telemedicine was. Therefore, these hypotheses were confirmed.

#### 4.4.1. The Scope of Internet Use for Healthcare Needs

The examinees were given a list of healthcare needs, and were asked to note if they used the internet for these needs. Table 2 below features the percentages of those examinees who responded “yes”.

Subsequently, a variable was constructed indicating the scope of internet use for healthcare needs by counting the positive answers for each examinee. The variable range was between 0 (does not use the internet at all for healthcare needs) and 8 (used the internet for all the uses mentioned). The average was 5.53 (SD = 1.40). Eight percent of the participants (*n* = 12) were in the range between 0–3, 36% of the participants (*n* = 57) were in the range between 4–5, and the rest were above 6 (*n* = 87, 56%). The relationships between this variable and the study variables were then examined and positive relationships of moderate significance were found between the scope of internet use for healthcare needs and eHealth literacy (r_p_ = 0.23, *p* < 0.01) and satisfaction with telemedicine (r_p_ = 0.25, *p* = 0.001).

#### 4.4.2. Gender Differences

Significant differences were found between the genders regarding their levels of e-literacy (t_(154)_ = 3.32, *p* = 0.001). Women had a higher degree of eHealth literacy compared to men (an average of 3.71 compared with 3.32). Significant gender differences were also found in relation to satisfaction with the use of telemedicine (t_(154)_ = 3.00, *p* < 0.01). Women had a higher degree of satisfaction compared to men (an average of 3.94 compared with 3.55). In addition, significant differences were found between the genders as to the scope of their use of the internet for healthcare needs (t_(154)_ = 4.33, *p* < 0.001). Women had a higher degree of satisfaction compared to men (an average of 5.86 compared with 4.88).

#### 4.4.3. The Differences between Education Levels

Examination of the differences between academics and non-academics showed that there were significant differences between them with regard to their level of e-literacy (t_(154)_ = 3.11, *p* < 0.01). Academics had a higher degree of eHealth literacy compared to non-academics (an average of 3.71 compared with 3.35). Significant differences between academics and non-academics were also found in relation to satisfaction with the use of telemedicine (t_(154)_ = 4.32, *p* < 0.001). Academics had a higher degree of satisfaction compared to non-academics (an average of 4.00 compared with 3.46). In addition, differences were also found in the scope of use of the internet for healthcare needs (t_(154)_ = 3.08, *p* < 0.01). Academics had a higher degree of satisfaction compared to non-academics (an average of 5.78 compared with 5.07).

### 4.5. A Regression Model for Forecasting Satisfaction with Telemedicine

Table 3 presents the results of a (multiple) linear regression model for forecasting satisfaction with telemedicine. The variables that were found to be significantly related to the dependent variable (age, sex, education, online vision literacy, and the scope of internet use for healthcare needs) were included in the model. In the preliminary model, gender and age did not provide a significant contribution to the model; therefore, they were removed from the final model. The final model appears below:

A significant regression was obtained (F_(154)_ = 14.63, *p* < 0.001), with an explained variance percentage of 22%. We can see that a higher level of eHealth literacy, an academic education, and greater use of the internet for healthcare needs lead to greater satisfaction with telemedicine.

## 5. Discussion

Telemedicine has developed considerably over the last two decades, making healthcare services considerably more accessible to outlying, peripheral areas. It has gained momentum in the wake of the COVID-19 pandemic due to pressure to reduce person-to-person interface and slow down the spread of the pandemic [25]. In Israel, there are marked gaps in the access to healthcare services in geographically peripheral areas [26]. This study illustrates the inherent potential in using the telemedicine tool during emergency periods, but also indicates that while telemedicine may be effective during a time of emergency, it may not be so when routine is restored. Therefore, it is important to better understand the characteristics of its use in order to maximize its potential.

The study participants demonstrated moderate to high levels of eHealth literacy. They knew how to search for medical information on the internet (85%) and how to use that information (65%), but were skeptical and suspicious about the reliability of using it, as only 36% felt confident about using the information from the internet to make health-related decisions. This finding is consistent with Farrugia et al. [27], who found that young people were skeptical about accessing online information regarding sexual health, explaining that today’s young people are presumed skeptical from the outset. Additional studies have arrived at similar findings (see for example [28,29]), and reliability evaluation has been mentioned as a key component in the process of searching for the healthcare information on the internet [30].

All the study participants used the internet for health-related needs. The study shows that they preferred to use the internet for “technical” needs, such as making a doctor’s appointment (92%), checking test results (86%), or asking for a repeat prescription (67%). However, regarding the need to engage in a treatment session (38%) or medical consultation (12%), the percentage figures were much lower. As can be found in other studies conducted during the COVID-19 era [31], the participants understood the advantages of telemedicine, such as improving waiting time and saving time as a whole (81%), the ease of use of a telemedicine app (70%), and improved accessibility (68%). However, in contrast to the findings in previous studies that showed a high level of satisfaction with online treatment sessions [31,32,33,34], in the current study, only 36% expressed satisfaction about the online sessions, and only 38% of the respondents who participated in treatment sessions on the internet felt that the online visits were identical in quality to in-person visits. Less than one half of them (45%) anticipated using telemedicine after the end of the pandemic. These findings are consistent with Sousa et al. [35], who found that “patients were satisfied with communication with providers; however, one half evaluated the online visit as inferior when compared to in-person visits”, and also with Horrell et al. [36], who found that 43% of the participants preferred to return to face-to-face, in-person meetings, whenever this would be possible in the post-COVID-19 era.

Although telemedicine is a simple way to maintain treatment continuity while reducing the risk of infection during a pandemic, a paradox has arisen from the COVID-19 experience. On the one hand, the COVID-19 pandemic has forced us into lockdowns and social distancing, thus increasing the use of telemedicine. On the other hand, this study shows that because of social distancing, patients are once again looking for the interpersonal relationship and human interaction with their physician, despite their awareness of the advantages of telemedicine. The COVID-19 era has been replete with tension, pressure, mental difficulties, and anxiety; when patients were in need of greater support, empathy, and a human touch, they were forced to forgo all of these. Moreover, while patients in studies conducted prior to the COVID-19 pandemic were able to choose between an in-person or online treatment session, during the COVID-19 pandemic crisis, online treatment was not the patient’s personal choice, but rather a requirement and an unavoidable necessity.

Contrary to the hypothesis, this study found no link between age and eHealth literacy, although previous studies have shown that younger users have used information available online in a more judicious manner than have older users [11]. There are a number of possible explanations for this finding. First, the survey was online meaning that, from the outset, there was a bias toward a more “technologically minded” population with access to internet services. Second, COVID-19 has promoted digitalization and has forced the more elderly population to also begin using online services, whether for shopping, virtual meetings with family members, or for access to healthcare services. Many of the elderly received the requisite technological support from their grandchildren, their neighbors, or volunteers who gladly rose to this challenge. The municipal authorities in Israel also came to the help of the elderly population to help with the digital challenge, as did private businesses, for example, the National Insurance Institution, which established a support group for all senior citizens seeking assistance and instruction on working with computers and/or smartphones as part of the “Computer for all Ages” program. The services included instruction provided to the elderly in their homes as well as instruction for pensioners within the community [37].

The negative correlation found between age and satisfaction with telemedicine is consistent with previous studies that showed the difficulties faced by the elderly with access to the internet and telemedicine [38,39], including studies conducting during the COVID-19 period [40,41,42]. The positive correlation between eHealth literacy and satisfaction with telemedicine was also supported by previous research studies [43,44].

Women tend to search for more healthcare information than men and use telemedicine more, as per Horrell et al. [36] who found that 50% of the women used telemedicine compared with 43% of the men, and expressed greater satisfaction with telemedicine [43]. On many occasions, it is the women in the family who are responsible for overall family health, so they are the ones who contact the physicians on behalf of other family members [45]. In addition, women have a longer life expectancy than men and tend to contract diseases, such as diabetes, hypertension, osteoporosis, and arthritis, etc.; therefore, they tend to engage in a much greater degree of contact with their family physicians and they consume more healthcare services, including those on the internet [46,47].

Examinees with an academic education expressed greater satisfaction and a broader scope of use of the internet for healthcare needs in relation to examinees without an academic education, as was the case in previous studies [48]. The educated generally tend to be more technologically minded, more pressed for time, and enjoy greater access to digital means.

In summary, the potential of telemedicine in the management of pandemics such as COVID-19 is great. Telemedicine can ensure the continuity of treatment and reduce the risk of infection [49]. The application of telemedicine reduces overcrowding in hospitals. Accordingly, this reduces the spread of viruses to patients and healthcare professionals [50]. Telemedicine should be applied in situations of isolation, locking and restriction of movement, especially in remote areas, or for those patients whose mobility is reduced [49]. However, there are still several obstacles to implementation. For example, data protection and patient privacy; ethical and legal standards regarding the medical profession; and disparities for access among ethnic and racial minorities, the elderly, and those on a low income, etc. [51].

### Study Limitations

The sample in this study is limited and is not representative of the adult population in the State of Israel. The study was conducted among patients in one clinic in a peripheral area and lacks a control group. The study tool was an online questionnaire so that, from the outset, there was a bias toward the younger and/or more technologically inclined population who possess computers or a smartphone and surf the internet. We may assume that a telephone survey or the manual distribution of questionnaires would have created a more precise picture.

## 6. Conclusions

It appears that the use of telemedicine is a significant tool also in geographically peripheral areas, and, consequently, it holds strategic significance for contending with existing gaps in the healthcare system. Young people and individuals with an academic education used more internet search strategies, and were more discerning and critical of the information available on the internet. The majority of these two groups were actually satisfied with telemedicine, and wanted to continue using it even after the end of the pandemic. Enjoying highly developed skills for eHealth literacy, this population is the main one to benefit from use of the internet and from digital age technology for medicine.

In contrast, the more elderly and those lacking an academic education generally tend to prefer continuing to use traditional communication channels to discuss their medical problems, such as in-person meetings with a clinician or telephone conversations, rather than virtual exchanges of information. Sometimes, members of these groups lack the technological know-how or the means to engage with health professionals on the internet. As a result of the lack of digital information among the more vulnerable strata of the population, who are in greater need of healthcare services, the gaps in healthcare are widening, and the resulting inequality between different sections of society is increasing.

eHealth literacy relates to a person’s ability to obtain, process, understand, and assimilate basic medical information when engaged in decision-making. As such, literacy is related to education, study, orientation, proficiency, control, and the ability to apply knowledge acquired. All these represent important skill sets in the 21st century. If these technologies were more user-friendly to patients, less cognitively loaded, and more understanding of the needs of elderly patients, it is possible that there would be greater use of telemedicine and additional exchanges of electronic information among the elderly as a whole. Today’s existing structure within the healthcare system and healthcare organizations creates a degree of complexity that limits the ability of the more elderly population, with weaker eHealth literacy, to use their services.

The behavior of healthcare consumers and of the health system and healthcare organizations under the limitations of the pandemic proves that it is possible to change a large number of components in the service model, such as a visit to the doctor or a consultation with him or her. There is a need to build instruction programs for those segments of the population who face difficulty in engaging in telemedicine, as well as programs to strengthen e-literacy as a whole, and eHealth literacy in particular. It is advisable to examine the perspectives and feelings of alienation associated with telemedicine on the part of both the patients and the clinicians. It would also be worthwhile to examine the scope of telemedicine use among people with limited mobility, and whether or not the use of telemedicine improves their quality of life.

## Figures and Tables

**Table 1:** Description of the sample characteristics.

Characteristic	Values	Overall Sample (*n* = 156)	
		*n*	%
Gender	Women	104	67
	Men	52	33
Country of birth	Israel	125	80
Level of education:	High school	31	20
	Professional post-school	23	15
	Academic	102	65
Religious level	Secular	92	59
	Traditional	45	29
	Religious	19	12
Existence of a health-monitoring app	Yes	123	79
Age group	19–30	68	44
	31–50	48	31
	Above 50	40	25

**Table 2:** Internet use for healthcare needs.

Use	Gave a Positive Response (%)
Reading an article or watching a video on medical-related topics	93
2. Making a doctor’s appointment	92
3. Consulting or reading about diseases, medications, or medical treatments	91
4. Checking test results	86
5. Receipt of updates on medical issues by email/text message	76
6. Request for a repeat prescription	67
7. Online treatment session with a doctor/nurse/dietician etc.	38
8. Attending medical forums for consultation	12

**Table 3:** Linear regression models for forecasting satisfaction with telemedicine.

Name of Variable	B	B	*p*
eHealth literacy	0.33	0.30 **	0.000
Education 0 = non-academic, 1 = academic	0.37	0.24 **	0.003
The scope of internet use for healthcare needs	0.23	0.17 *	0.025
R^2^	0.22		
_Adj_ R^2^	0.21		
*N*	156		

* *p* < 0.05, ** *p* < 0.001.

## Data Availability

The data that support the findings of this study are available from the corresponding author.

## Appendix A

### Questionnaire

Dear Sir/Madam:

The purpose of this survey is to become familiar with the public’s attitude toward telemedicine conducted via the internet. Filling out the questionnaire is on a volunteer basis. The survey is anonymous and we wish to point out that all your answers remain confidential. The data will be processed in a group form alone. The instructions are written in the masculine for the purpose of convenience, but are intended for both women and men. For questions please contact Dr. Keren Dopelt: dopelt@bgu.ac.il.

Thank you for your cooperation.

Please state if during the last six months you have carried out the following actions on the internet:

	**(1) Yes**	**(2) No**
(1) You have read an article or watched a video on medical-related topics		
(2) You have received by email/text message updates on medical issues		
(3) You have consulted or read about diseases, medications or medical treatments		
(4) You have checked test results		
(5) You have made a doctor’s appointment		
(6) You have asked for a repeat prescription		
(7) You took part in an online treatment session with a doctor/nurse/dietician etc.		
(8) You attended medical forums for consultation		

To what extent was/did the use you made of telemedicine via the internet:

	**(1) Not At All**	**(2) To a Small Extent**	**(3) To a Moderate Extent**	**(4) To a Large Extent**	**(5) To a Very Large Extent**	**(6) Irrelevant**
(1) Improve your access to healthcare services						
(2) Appropriate for your healthcare needs						
(3) An excellent solution for the COVID-19 period						
(4) The apps are easy to use						
(5) Save a lot of time and waiting in line						
(6) I am satisfied with my experience of a treatment session with medical staff via the internet						
(7) The online visits were identical in quality to in-person visits						
(8) I tried to enter an online treatment session but without success						
(9) I will continue to use telemedicine services for treatment sessions even after the pandemic						
(10) Overall, I am satisfied with the telemedicine services						

To what extent do you:

	**(1) Not At All**	**(2) To a Small Extent**	**(3) To a Moderate Extent**	**(4) To a Large Extent**	**(5) To a Very Large Extent**
(1) Know how to use the internet to respond to health-related questions					
(2) Know how to find useful healthcare information on the internet					
(3) Know how to use the healthcare information you found on the internet					
(4) Know how to evaluate the healthcare information you find on the internet					
(5) Feel safe to use the information on the internet in order to make health-related decisions					
(6) Consume more healthcare related information on the internet since the outbreak of the COVID-19 pandemic					
(7) Look for information on the internet about the COVID-19 vaccine					

What is your year of birth? _________________

What is your gender? (1) Male (2) Female (3) I prefer not to answer

Level of education: (1) Elementary school/junior high (2) High school (3) Professional post-school (4) Academic

Where were you born? (1) Israel (2) Former Soviet Union states (3) Other: ____________________

If you were not born in Israel, in what year did you make aliyah? _______________________

Does your mobile phone have apps that help you manage or monitor your state of health (apps of health maintenance organization, tests, various health-related parameters)? (1) Yes (2) No (3) I do not know (4) I do not possess a smartphone

How religious are you? (1) Secular (2) Traditional (3) Religious (4) Ultra-Orthodox
